# Making use of equity sensitive QALYs: a case study on identifying the worse off across diseases

**DOI:** 10.1186/1478-7547-12-16

**Published:** 2014-07-23

**Authors:** Frode Lindemark, Ole Frithjof Norheim, Kjell Arne Johansson

**Affiliations:** 1 Department of Research and Development, Haukeland University Hospital, Jonas Liesvei 65, 5021 Bergen, Norway

**Keywords:** Priority setting in health, Quality-adjusted life years, Severity, Healthcare rationing, Equity

## Abstract

**Background:**

Resource allocation decisions currently lack standard quantitative methods for incorporating concerns about the worse off when analysing the cost-effectiveness of medical interventions.

**Objective:**

To explore and demonstrate how to identify who are the worse off without a new intervention by measuring lifetime Quality-Adjusted Life Years (QALYs) for patients across different conditions, and compare the results to using proportional shortfall of QALYs.

**Methods:**

Case study of eight condition-intervention pairs that are relevant to priority setting in Norway; childhood deafness (unilateral cochlear implant), unruptured cerebral aneurysm (coiling), morbid obesity (RY gastric bypass), adult deafness (unilateral cochlear implant), atrial fibrillation (catheter ablation), hip osteoarthritis (hip replacement), rheumatoid arthritis (TNF inhibitor) and acute stroke (stroke unit). We extracted prospective QALYs without and with new interventions from published health technology assessments and economic evaluations.

**Results:**

Among the eight cases, the lifetime QALY method and the proportional shortfall method yielded conflicting worse-off rank orders. Particularly two conditions had a substantial shift in ranking across the applications of the two methods: childhood deafness and acute stroke. Deaf children had the lowest expected lifetime QALYs (38.5 without a cochlear implant) and were worst off according to the lifetime approach, while patients with acute stroke had the second-highest lifetime QALYs (76.4 without stroke units). According to proportional shortfall of QALYs, patients with acute stroke were ranked as worse off than deaf children, which seems counterintuitive.

**Conclusion:**

This study shows that it is feasible to identify who are the worse off empirically by the application of lifetime QALYs and proportional shortfalls. These methods ease further examination of whether there is a true conflict between maximization and equity or whether these two concerns actually coincide in real world cases. It is yet to be solved whether proportional prospective health losses are more important than absolute shortfalls in expected lifetime health in judgements about who are worse off.

## Background

Health economic evaluations and comparative effectiveness analyses are increasingly used to inform healthcare priorities [1,2]. Cost-effectiveness analysis (CEAs) with Quality-Adjusted Life Years (QALYs) is the dominant method for determining which health interventions maximize health the most. The underlying assumption of such methods, that a QALY has the same worth regardless of who gets it, i.e. severity of illness, age, or social deprivation, is a well-known ethical controversy [3].

In political philosophy, there is an extensive literature on general arguments in favour of prioritising those who are worse off, e.g. in terms of income, education, social integration, opportunities, capabilities or health [3]. In the field of health, to be worse off may refer to current suffering or disability, expected future health losses, expected lifetime health, or simply to having the most severe health problem. More precision is needed in actual policy decision-making where large populations and opportunity costs are at stake. Who is worse off: Those in worse health at the time of intervention or those who will be in worse health if not treated, or those who will have the worst lifetime health if not treated [4]?

As regards concerns for worse off in priority setting of health interventions, discrete choice experiments, policy documents and public debate in countries like Norway, Sweden and the Netherlands have emphasized the relevance of current severity and/or expected health losses at the time of intervention [5-10]. This particular view gives extra weight to health gains to those with greater current severity and expected future loss of quality and/or length of life due to disease. In general, this position disregards age and past loss of quality of life as relevant, or leaves these to be treated as separate concerns [11]. In contrast, recent discrete choice studies indicate that people‘s preferences are also reflected by the view that health gains to those with less expected total lifetime should have priority [12,13]. This opposing view, that health care resources should be allocated so as to reduce inequalities in total lifetime health, is supported in previous works by Williams and Norheim and Asada [14,15].

We need a better understanding of how different views on prioritising the worse off can be operationalised and quantitatively integrated into models of cost-effectiveness analysis [16,17]. Dutch researchers and policymakers have proposed to operationalise concerns for the worse off by adjusting the cost-effectiveness threshold according to the proportional shortfall of QALYs caused by the condition in question. Proportional shortfall of QALYs is a severity measure identifying the worse off as those with the greater current and expected health losses relative to a remaining lifetime spent in perfect health [9,18]. Ottersen recently provided a range of arguments for giving priority to those with fewer lifetime QALYs [19]. In principle, a measure of lifetime QALYs for a patient group would take into account the average health losses due to disease, both in terms of quality of life and longevity, over the entire lifetime, and is thus a quantification of worse off with respect to lifetime health.

In this article, we identify the worse off by measuring lifetime QALYs and proportional shortfall of QALYs for a realistic set of eight condition-intervention pairs with data from published health economic evaluations, and we illustrate how these measures offer ethically relevant supplementary information to the effectiveness and costs of interventions. The aim is to facilitate an explicit and empirically based debate on the relevance of expected lifetime health compared to other alternatives by showing how different criteria would lead to different rankings of conditions, and therefore, different priorities.

## Methods

### QALY information and definitions

QALYs are used as a generic measure of health outcomes in health economic evaluations. A QALY can be understood as the equivalent of one life year in perfect health. It is the product of the time spent in a health state and a quality-adjustment weight ranging from 0 (death) to 1 (perfect health), reflecting people‘s preferences for the health state [20].

**Figure 1:**
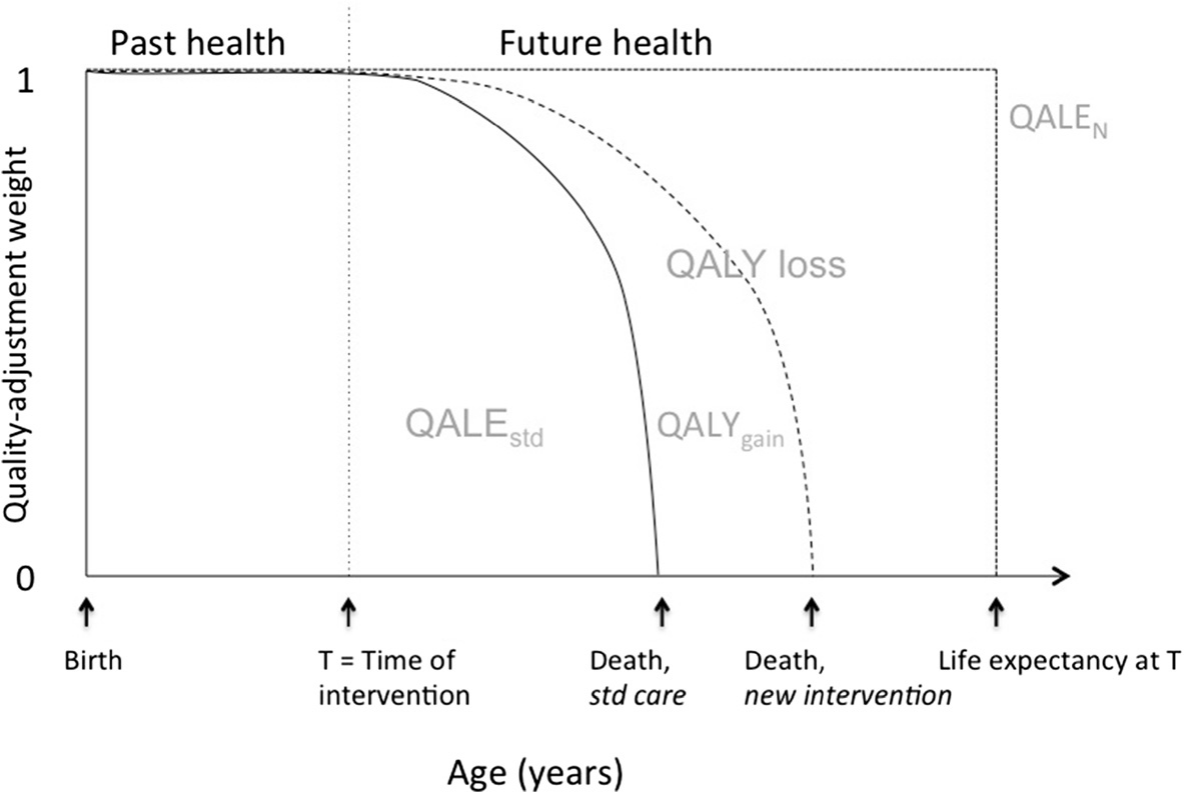
Representation of the QALY concept in a lifetime perspective.

To determine the lifetime QALYs and proportional shortfall of QALYs for patients with a given condition without the new intervention, we need three variables (see Figure 1). First, the mean age of the patient group at the time of intervention (past health). Ideally we would need these previous life years to be quality adjusted, but we found no studies reporting such adjustments. We therefore assumed that all patients had perfect health until the time of the interventions. Second, the remaining Quality-Adjusted Life Expectancy without the new intervention (QALE_std_, the starting point of QALE_std_ is the time when the intervention starts (T)), and the ending point is the time of death without the new intervention). Third, the average number of remaining QALYs in absence of illness for people of the same age as the patient group (QALE_N_). In addition, we report the expected QALYs gained (QALY_gain_) by each intervention, which is simply the estimated increase in QALYs by the new intervention compared to standard care.

Lifetime QALYs is estimated by the sum of past health (in this analysis we use average age of the patient group as a proxy for past health) and average prospective health (QALE_std_) per patient. Proportional shortfall of QALYs is estimated by calculating the expected future loss of QALYs due to disease relative to a perfectly healthy life expectancy (QALE_N_).

Often, QALE_std_ and QALY_gain_ are discounted and generated for a shorter than lifetime horizon. QALE_std_ is not always reported in published CEAs, even if this is a key output parameter in most CEA models. In this analysis, we extracted QALE_std_, QALY_gain_, and the mean age of the patient group at the time of intervention from studies of eight example cases. QALE_N_ was taken from a Norwegian life table (2010) unless stated otherwise. We report and compare lifetime QALYs, QALY loss, proportional shortfall of QALYs, and QALY_gain_ for all example cases.

### Selection of cases and search strategies

First, we selected eight condition-intervention pairs as example cases. These cases were chosen because they 1) are relevant to priority setting in Norway, and 2) illustrate properties of the measures of lifetime QALYs and proportional shortfall of QALYs. The condition-intervention pairs are relevant as they differ with regard to the average age of the patients, chronicity, impact on longevity and quality of life, and the effectiveness, cost, and frequency of the interventions (Table 1). Deciding between them potentially involves value judgments regarding whether to prioritise younger vs. older patient groups, acute vs. chronic diseases, few vs. many patients, highly effective vs. less effective interventions, and high-cost vs. low-cost interventions. The decision involves judgments about how to balance these different categories, all of which are relevant to the fair allocation of scarce healthcare resources [21,22].

**Table 1:** Condition-intervention pairs ranked in order of gain in QALYs

**Patient group**	Age*	**QALE**_**std**_**†**	**QALY**_**gain**_**‡**		**Priority relevance**	**Source**	**References**
**Childhood deafness**	8	30.5	10.7	(3.9)	High-cost, low-volume health care intervention. To date, relatively few patients have been considered eligible for a cochlear implant. Cochlear implantation has become an established routine treatment option for profoundly deaf adults and children who do not benefit from acoustic hearing aids both in Norway and around the world.	HTA	Bond 2009 [25]
(unilateral cochlear implant vs hearing aid and waiting list for implant)							
**Unruptured cerebral aneurysm**	50	23.9	6.4	(3.6)	High-risk patient with symptomatic aneurysm produces subarachnoid haemorrhage (SAH) with substantial rate of mortality (30-60%) and permanent disability (15-30%). Over the years, there has been debate about which unruptured aneurysm to treat.	CUA	Johnston 1999 [26]
(Coiling vs. no treatment)							
**Morbid obesity**	48	28.2	5.2	(2.3)	Increasing public health problem in Norway and elsewhere. Increased risk of premature death and reduced quality of life due to obesity-related co-morbidities. Potential demand for bariatric surgery is greater than availability.	HTA	Klarenbach 2010 [27]
(RY gastric bypass vs. lifestyle modification: diet and exercise medical counselling)							
**Adult deafness**	50	14.0	4.2	(2.4)	See childhood deafness	HTA	Bond 2009 [25]
(Unilateral cochlear implant implant for adult)							
**Atrial fibrillation**	52	17.3	2.3	(1.4)	Uncertainty about the intervention‘s long-term effects on stroke risk, mortality and QoL, but already established as an attractive alternative to drug-refractory AF in symptomatic patients with recurrent AF. Waiting list 0.5-1 year in Norway. Causes patients to pursue treatment abroad, some at their own cost.	CUA	McKenna 2009 [28]
(Catheter ablation vs. antiarrhythmic drug therapy							
**Hip osteoarthritis**	63	19.8	1.3	(0.9)	High-volume, relatively high-cost intervention. Five thousand hip arthroplasties per year. Half of the adult population at risk.	CUA	Rasanen 2007 [29]
(Hip replacement vs. nonoperative approach)							
**Rheumatoid arthritis**	55	6.1	1.3	(1.0)	20 000–30 000 patients in Norway. Lifelong burden of pain, discomfort and physical impairment; the years of life lost are estimated to be 5–7 years. In Norway, at least one DMARD has to be tried before prescribing biological agents such as TNF inhibitors on the grounds of the higher cost of biological agents, although combination therapy with a TNF inhibitor is more effective in treating rheumatoid arthritis.	HTA	Chen 2006 [30]
(TNF inhibition + methotrexate vs. Methotrexate)							
**Acute stroke**	70	6.4	0.5	(0.3)	Approximately 15 000 cases annually in Norway and is the third most common cause of death; it is a major cause of severe disability and accounts for a significant proportion of healthcare spending. Over the past years, there has been a focus on developing stroke units at hospitals around the country.	HTA	Hamidi 2010 [31]
(Stroke unit vs. general ward)							

*Average age (years) at the time of intervention.

†Expected remaining quality-adjusted life years given a certain disease with standard care. Undiscounted data. Utilities expressing the current severity can be found in Additional file 2.

‡Undiscounted data. The discounted gain in QALYs is shown in brackets. Note that the annual discount rates vary between the source studies [see Additional file 2]. Ranking according to the discounted gains in QALYs would yield a different order.

Abbreviations: *AF* atrial fibrillation, *CUA* cost-utility analysis, *DMARD* disease-modifying antirheumatic drug, *QoL* quality of life, *HTA* health technology assessment, *QALY* quality-adjusted life year, *RY* roux-en-y, *TNF* tumour necrosis factor.

Second, we used an evidence-based approach and searched for Health Technology Assessments (HTAs), systematic reviews of clinical effectiveness, and cost-effectiveness analyses for selected conditions and interventions, as recommended for rapid reviews and mini-HTAs [23,24]. An additional file shows this in more detail [see Additional file 1]. We identified one economic evaluation for each of the eight condition-intervention pairs from which we extracted QALY data. Additional file 2 provides information about the QALY construction in the source studies. Studies that did not have information on QALE_std_ were excluded from this analysis. If they were not published, we received undiscounted data upon request from the author of the original published study (Table 1) [25-31].

## Results

**Figure 2:**
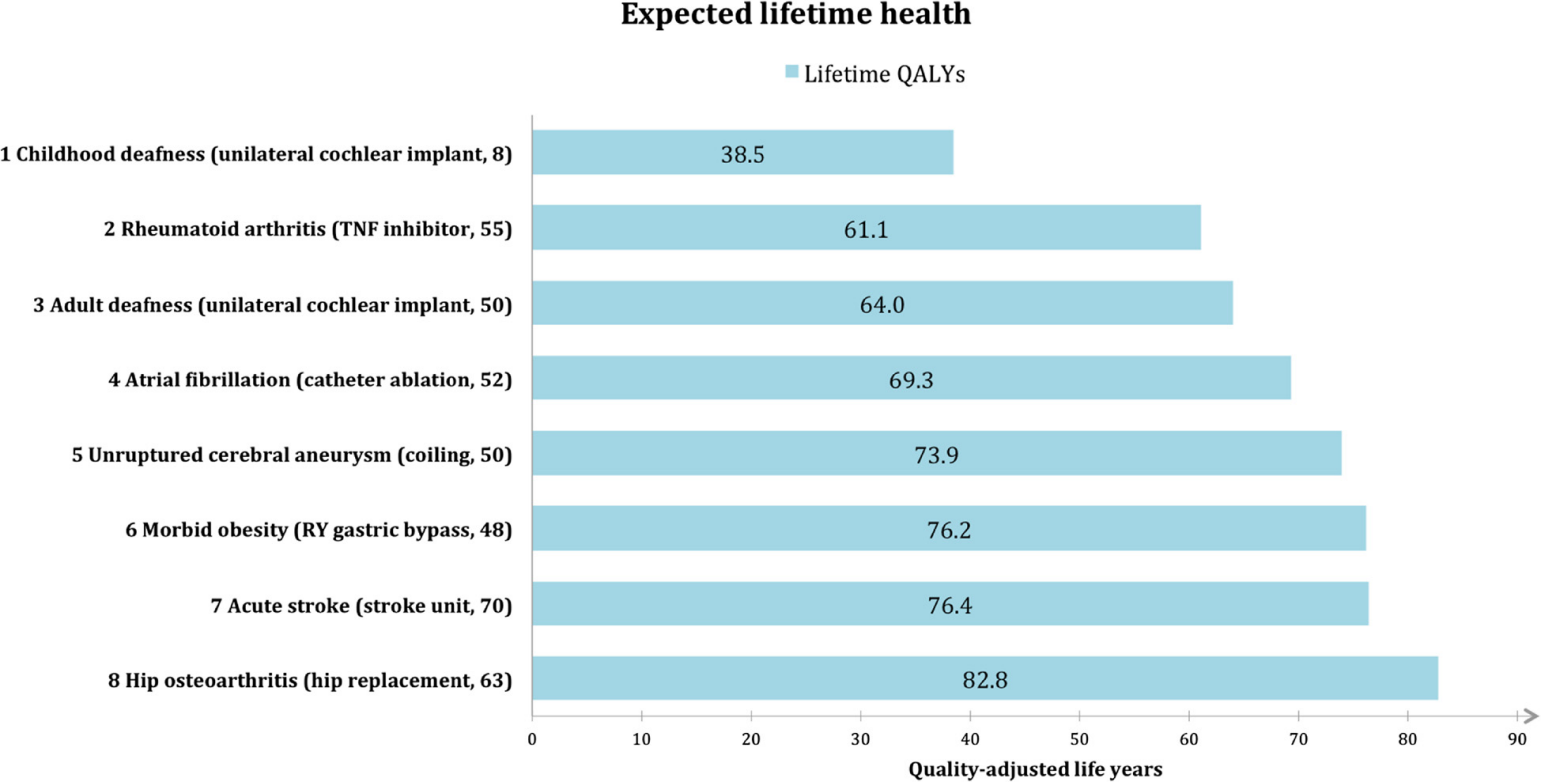
**Ranking according to lifetime QALYs (age + QALE**_**std**_**) for eight condition-intervention pairs.** Childhood deafness had the worst prognosis (top) and hip osteoarthritis had the best prognosis (bottom) from a lifetime perspective. Abbreviations: QALE_std_, remaining quality-adjusted life years given a certain disease treated with standard care; QALY, quality-adjusted life year; RY, roux-en-y; TNF, tumour necrosis factor.

On a lifetime QALY account, the eight conditions would be ranked from the worst-off to the best-off as follows: patients with childhood deafness (38.5 lifetime QALYs) rheumatoid arthritis (61.1 lifetime QALYs), adult deafness (64.0 lifetime QALYs), atrial fibrillation (69.3 lifetime QALYs), unruptured cerebral aneurysm (73.9 lifetime QALYs), morbid obesity (76.2 lifetime QALYs), acute stroke (76.4 lifetime QALYs), and hip osteoarthritis (82.8 lifetime QALYs) (Figure 2).

**Figure 3:**
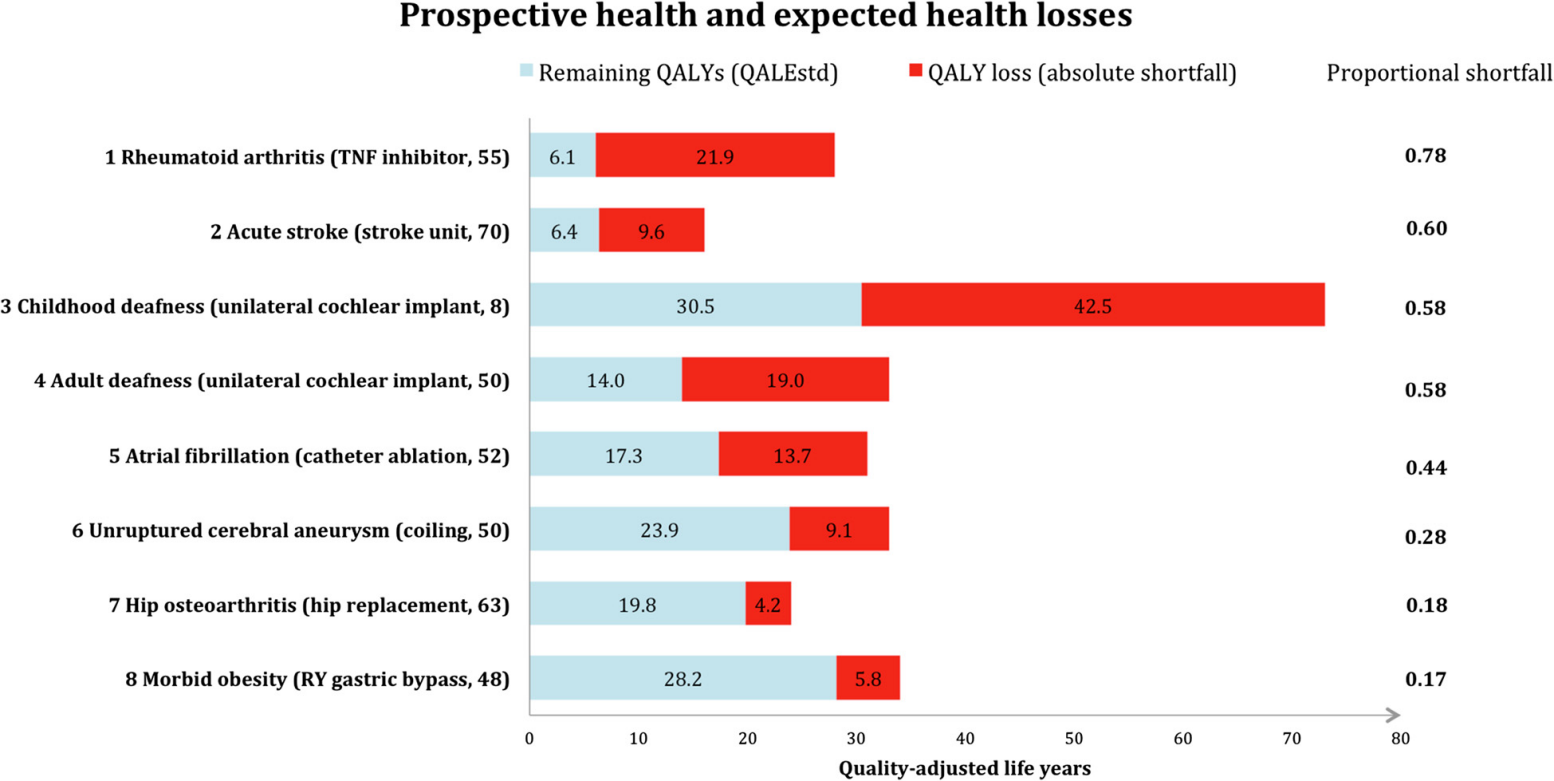
**Ranking of conditions according to proportional shortfall of QALYs.** Patients with rheumatoid arthritis and acute stroke were worst off and patients with hip osteoarthritis and morbid obesity were best off. For each condition, the total length of the bar represents the remaining QALYs in absence of illness (QALE_N_). The red part of the bar represents the absolute shortfall of QALYs due to illness. Abbreviations: QALE_std_, remaining quality-adjusted life years given a certain disease treated with standard care; QALY, quality-adjusted life year; RY, roux-en-y; TNF, tumour necrosis factor.

**Figure 4:**
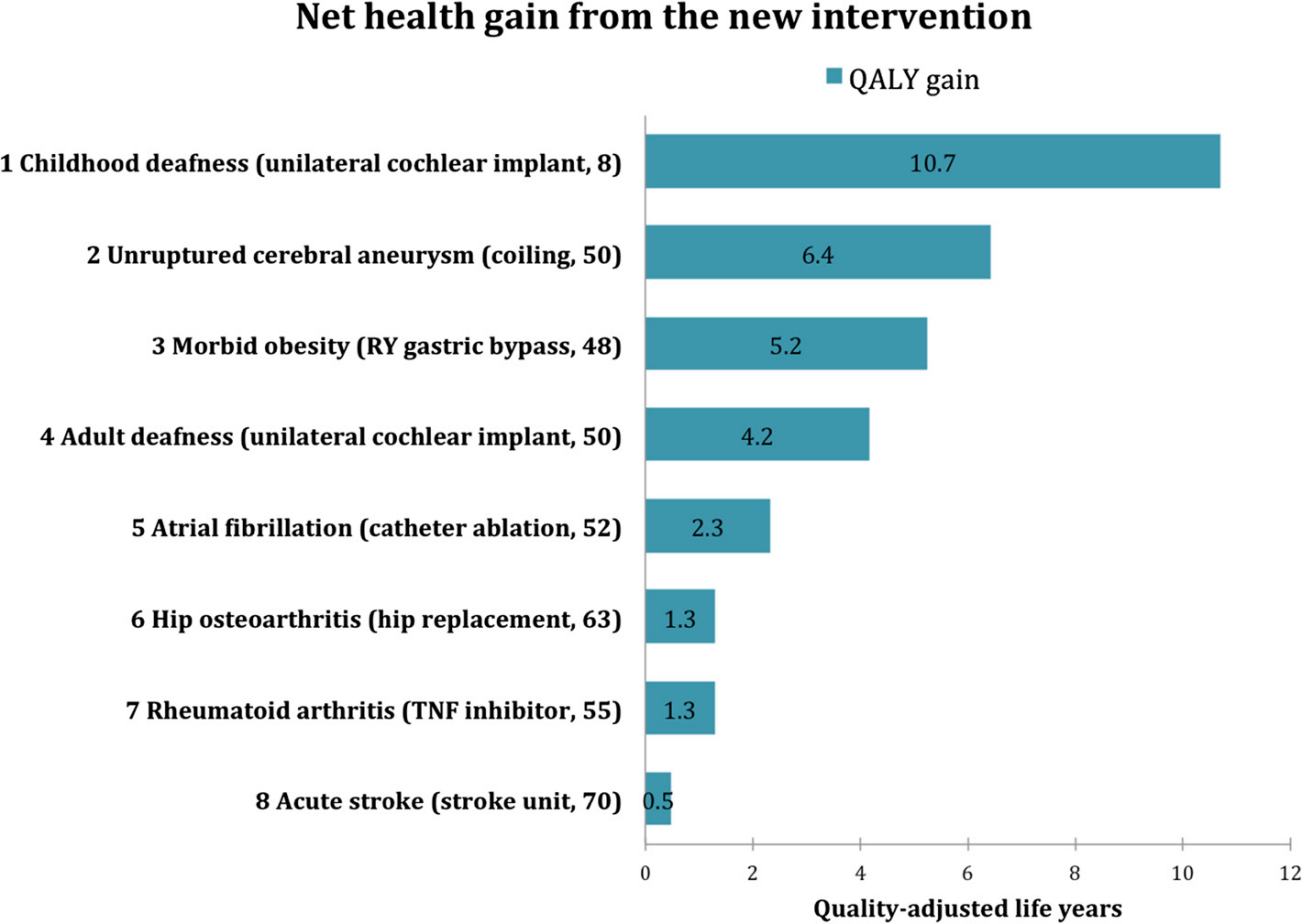
**The condition-interventions pairs ranked according to the net health gain from the intervention.** Unilateral cochlear implant to deaf children was most effective, and stroke units for acute stroke victims were least effective. Abbreviations: QALY, quality-adjusted life year; TNF, tumour necrosis factor; RY, roux-en-y.

According to the measure of lifetime QALYs, the worst-off patients were those with a chronic condition starting early in life, as childhood deafness, even though deafness affects only quality of life and has no impact on mortality. We found that patients with acute stroke – which is generally considered a very severe disease, often causing both premature death and severe disability – were comparatively better off because this group, on average, lose QALYs late in life.Proportional shortfall of QALYs placed childhood deafness third (proportional shortfall = 0.58), behind rheumatoid arthritis (proportional shortfall = 0.78) and acute stroke (proportional shortfall = 0.60) (Figure 3). Among the best off, patients with morbid obesity (proportional shortfall = 0.17) were better off than patients with hip osteoarthritis (proportional shortfall = 0.18) according to the proportional shortfall as compared to the lifetime QALYs approach.Unilateral cochlear implants for childhood deafness yielded the highest gain (10.7 QALYs), and stroke units for acute stroke victims yielded the lowest gain (0.5 QALYs) (Figure 4).Note especially two condition-intervention pairs in Figure 2 through Figure 4: (A) rheumatoid arthritis/TNF inhibitor and (B) morbid obesity/gastric bypass. According to both lifetime QALYs and proportional shortfall of QALYs, A is worse off than B, whereas the gain in QALYs is higher for B than for A. Below, we discuss why this is ethically relevant.

## Discussion

Our results confirm that it is possible to use the existing literature to estimate lifetime QALYs and quantitatively compare groups with different conditions to identify who are worse off, and to compare the lifetime account with the proportional shortfall about prospective severity. For example, we found that lifetime QALYs are lower for childhood deafness and rheumatoid arthritis than for acute stroke and hip osteoarthritis. A proportional shortfall approach yields a different ranking, the main difference being that acute stroke victims become worse off, second only to rheumatoid arthritis. This information is highly relevant for transparent and evidence-based political discussions on how to assign higher weight to health gains for those who are worse off.

Resources will be allocated to different patients groups, depending on whether extra weights are given to those who can expect to attain fewer QALYs over their lifetime versus to those who can expect greater relative future loss of QALYs. To our knowledge, this is the first study based on data from published economic studies to consider the worse off as being those with fewer lifetime QALYs, and which compares this lifetime health approach with the prevailing view of defining the worse off in terms of relative losses in current and future health.

Most proposed definitions of the worse off have a future-oriented perspective [5]. The measure of lifetime QALYs is persuasive since, in principle, absolute differences in past and future health is taken into account. The length and quality of life lived before time of intervention may be relevant in judgements about who are worse off, and it is not obvious that these concerns should be disregarded. For example, early onset of disease is the main reason why deaf children achieve fewer lifetime QALYs than deaf adults (annual quality of life losses are assumed to be similar for both groups, an additional table provide quality-adjustment weights) [see Additional file 2]. Children who are born deaf will suffer from lack of language skills throughout their entire life if left untreated. Despite substantial prospective impairment in quality of life among adults who develop profound deafness, they have developed normal language skills earlier in life, and are less prone to all of the negative lifelong effects of early deafness [32]. Consequently there are good reasons to give higher priority to quality-improving interventions, such as the cochlear implant, to the deaf child than to the deaf adult. Lifetime QALYs identifies the child as worse off. Proportional shortfall of QALYs does not discriminate between the two groups. Past health would have to be treated as a separate concern if this was to be taken into account in the proportional shortfall approach (Figures 2 and 3).

Even if deaf children will suffer from lack of language skills their entire life, they will also, however, adapt to their disability and learn skills required for functioning without hearing at a younger age. The lifetime QALY approach therefore entails the same adaptation problems as health state valuations in standard QALY applications [33]. Discrete choice techniques may adjust for adaptation over time by obtaining health state valuations from the general public rather than directly from patients [34]. Nevertheless, it is critical what respondents are asked in preference elicitation studies and there is need for additional empirical and normative work to fully understand the complexities of adaptation over a lifetime [35].

Absolute shortfall of future health (QALY loss) is a measure suggested by the UK Department of Health and National Institute for Health and Care Excellence to be used in weighting the societal wider impact of conditions, while using proportional shortfall in weighting the burden of illness, in value-based assessment of interventions [36]. We define absolute shortfall of QALYs as the expected future loss of QALYs from a condition, i.e. the absolute difference between the QALY expectancy in absence of illness (QALE_N_) and the remaining QALYs with standard care at the time of intervention. The lifetime QALY approach treats lifetime QALY attainments and lifetime QALY loss as providing the same information. Values of these two lifetime measures are inversely correlated, and rank orders will not change if QALE_N_ were the same for all interventions.

Arrow has argued that equivalent formulations of a choice problem should yield the same preference order [37]. In principle, a lifetime QALY approach would therefore yield the same preference order for both QALY attainment and QALY shortfall if QALE_N_ were identical for all age groups. Nevertheless, people are typically more averse to losses than equivalent gains [38]. Whether people actually are less averse to QALY attainments than equivalent QALY shortfalls over a lifetime remains unclear. More empirical work is needed to test preferences of the general public on these matters.

Proportional shortfall of QALYs discriminates between conditions with respect to relative differences in prospective health. The measure identifies the worse off as those who stand to lose the largest fraction of their health potential [9]. The rationale appears to be that people of all ages is entitled to fulfil the health potential they had reason to expect in absence of illness. When we compare the rankings of conditions according to lifetime QALYs and proportional shortfall, we find that the proportional shortfall measure hardly discriminates between childhood deafness, adult deafness, and acute stroke (Figure 3), despite the fact that the number of healthy life years for these patient groups differs considerably: the lifetime QALYs were 38.5, 64.0, and 76.4, respectively (Figure 2). The measure of lifetime QALYs distinguishes between conditions with respect to the individual burden of disease over the total lifetime.

We argue that underlying concerns about fair distribution of health are better captured by directing resources towards those with fewer lifetime QALYs rather than to those with a higher proportional shortfall of QALYs, because the former would seek to reduce inequalities in lifetime health, while the latter would seek to reduce inequalities in the future health potential only [15,39].

Our results elucidate the balance between the worst condition and the most effective intervention. Usually, both concerns point in the same direction. For example, among the eight cases, our results show that cochlear implantation in children is the most effective intervention and that the deaf child is also among the worst off. However, sometimes, one intervention will maximise health and increase inequality. In the comparison between the two condition-intervention pairs (A) rheumatoid arthritis/TNF inhibitor and (B) morbid obesity/gastric bypass (Figure 3), a decision to offer gastric bypass first and decline to offer the TNF inhibitors would maximise average individual health outcomes, but it would also increase the inequality in lifetime health, and the inequality in future health potential, between patients with rheumatoid arthritis and obese patients. In cases where there is a conflict between concerns for the worse off and the effectiveness of the interventions, decision makers must balance competing concerns or rely on fair procedures [3]. Decision makers could give greater weight to the TNF inhibitors because of fairness considerations: patients with rheumatoid arthritis represent the worse off group of the two.

Our study has some limitations. First, only the time aspect of past health is captured by the method we used to calculate lifetime QALYs. Past differences in quality is not taken into consideration since we lack data on past quality of life for the various conditions. Our sources, conventional CEAs, start calculating QALYs at the time of intervention. Among the eight conditions, morbid obesity is likely to be ranked relatively higher if past suffering were taken into account. Morbidly obese patients who are eligible for surgery at the average age of 48 would probably have suffered due to obesity and obesity-related comorbidities over many years, often since adolescence or childhood [27]. The time elapsed between disease onset and the time of intervention would be much shorter for rheumatoid arthritis, atrial fibrillation, and hip osteoarthritis. Knowledge about quality of life losses in this period would result in fewer lifetime QALYs, but it is not obvious that this would change their rankings. The inclusion of past quality of life losses is practically challenging, and theoretically perhaps the most controversial implication of the lifetime health account [19].

Second, few studies have reported lifetime QALYs, so there is a shortage of available data. Third, the comparability of QALY data across various studies and analytical decision models is impeded by the variations in model structure complexity, varying time horizons, and varying discount rates. Additional file 2 shows key model assumptions in the source studies. Our results are based on undiscounted data. The discount rates used in the base cases of the source studies differ. The reasons to discount health outcomes at a specific rate are usually independent of the conditions and interventions that are being assessed. Therefore, using discounted QALY profiles may affect their relative size, and consequently our rankings, without good reason. There are good fairness reasons to argue that a life year in the past, present, or future should have the same value, but there is no agreement about the role of discounting [40]. We decided to include only studies that estimated effects over a lifetime horizon. However, investigators seem to be reluctant to pursue a lifetime analysis [see Additional file 1]. The extrapolation of effects over a lifetime involves uncertainty, but textbooks and guidance on health economic evaluations generally recommend that a lifetime horizon be applied [41]. Fourth, the validity of the results is subject to limitations in terms of the QALY methodology. Standard QALY calculations involve rather strong underlying assumptions [20]. Some uncertainty is attributed to the quality-adjustment weights [42]. The results are probably sensitive to the quality-adjustment weights used in the source studies [see Additional file 2]. Our approach requires that QALYs be generated in as consistent a manner as possible. The QALY consensus group has pointed out the need to develop a reference method for estimating QALYs [43].

## Conclusion

In this study we have quantified worse-offness with respect to lifetime health by measuring total lifetime QALYs, and compared the results to using proportional shortfall of QALYs to identify the worse off. Our results illustrate that the lifetime QALY measure identifies those who stand to attain the lower number of healthy life years over the entire lifetime as worse off. Absolute shortfall of QALYs yields similar results. Proportional shortfall of QALYs, which focuses on relative current and prospective health losses, is not sensitive to large absolute differences in expected lifetime health. The absolute measures reveals, while the relative measure conceals, the size of the health loss. The lifetime QALYs approach is based on the principle of equal lifetime health. The relative shortfall approach is based on the notion that people of all ages have a legitimate claim to fulfil their health potential. The lifetime QALYs approach challenges the prevailing view that emphasizes prospective severity and could enhance fair prioritisation across patient groups because it helps identify cases where resources should be directed towards interventions for those with the least expected lifetime health.

## Competing interests

All authors have completed the Unified Competing Interest form at http://www.icmje.org/coi_disclosure.pdf (available on request from the corresponding author) and declare: no support from any organisation for the submitted work; no financial relationships with any organisations that might have an interest in the submitted work in the previous 3 years; no other relationships or activities that could appear to have influenced the submitted work. This article is part of the Western Norway Regional Health Authority (WNRHA) research programme “Priority setting across clinical specialties”. OFN (Professor) leads the research programme. KAJ (Senior Researcher) and FL (PhD candidate) are employed in the programme. The authors declare that they have no competing interests.

## Authors‘ contributions

All authors were involved in the conception and design of the study, the selection of cases, the critical revision, and the final approval of the submitted version of the manuscript. FL and KAJ developed the eligibility criteria for including studies and analysed the data. FL performed the literature search. KAJ is the guarantor.

## Supplementary Material

Additional file 2 Key model assumptions of source studies. Click here for file

Additional file 1 Search strategies. Click here for file
