# Supplementary material for: Making use of equity sensitive QALYs: a case study on identifying the worse off across diseases
Source: Cost Eff Resour Alloc. 2014 Jul 23;12:16. doi: 10.1186/1478-7547-12-16 (PMC4118218; doi:10.1186/1478-7547-12-16)
Supplement: Additional file 2 — Key model assumptions of source studies. [file 1478-7547-12-16-S2.pdf]

Additional file 2. **Key model assumptions of source studies**

Patient group	QALY construction				Discount rate	References
	Quality-adjustment (Q-a)			Mortality		
	Health state	Q-a weight	Instrument			
**Childhood deafness*** (Unilateral cochlear implant vs. hearing aid and waiting list for an implant for a child, age 8 years)	Deaf (Preimplantation)<br>Deaf (Postimplantation)	0.421<br>0.653	Adapted HUI-3	Assume life-long effect of the device until the expected age of death for 1 year olds at 80 years. Years of life lost due to deafness and the evaluated technology were zero.	3.5 %	Bond 2009[25]
**Unruptured cerebral aneurysm*** (Coiling vs. no treatment, mean age 50 years)	Healthy<br>Dead<br>Permanent mild disability<br>Permanent moderate/severe disability<br>Untreated aneurysm (No symptoms)<br>Untreated aneurysm (mild symptoms)	1<br>0<br>0.76<br>0.25<br>1-5 x (rate of rupture)<br>0.84	 <br>TTO and SG<br>TTO and SG<br>estimate<br>HUI-2	US age- and sex-specific background mortality. Modification by SAH yearly rates and case-fatality, procedural risks and disabled patients.	3%.	Johnston 1999[26]
**Morbid obesity*** (RY gastric bypass vs. lifestyle modification: diet and exercise medical counselling, mean age 48 years)	BMI 28 (reference state)<br>BMI change +/- 1 unit (Diabetic)<br>BMI change +/- 1 unit (Nondiabetic)	0.71<br>+/- 0,017<br>+/- 0,0285	VAS, TTO	Mortality among obese patients in Canadian life tables. The data were modified by perioperative outcomes, and the relative risk of death after surgery was compared with usual care. After ten years, the mortality was assumed to be identical for both strategies.	5%.	Klarenbach 2010[27]
**Adult deafness*** (Unilateral cochlear implant vs. hearing aid and waiting list for an implant for an adult, age 50 years)	Deaf (Preimplantation)<br>Deaf (Postimplantation, gradual decline after age 55 years)	0.433<br>0.63	HUI-3	Assume life-long effect of the device until the expected age of death for 50 year olds at 82 years. Years of life lost due to deafness and the evaluated technology were zero.	3.5 %	Bond 2009[25]
**Atrial fibrillation*** (Catheter ablation (RFCA) vs. antiarrhythmic drug (AAD) therapy, mean age 52 years)	UK general population (NSR)<br>*NSR given RFCA*<br>*NSR given AADs*<br>*AF given RFCA*<br>*AF given AADs*<br>*pulmonary toxicity*<br>*nonpulmonary toxicity or bleeding event (days of perfect health lost)*<br>Disabled stroke<br>Nondisabled stroke<br>Combined stroke (30.9% disabled)	Baseline<br>*0*<br>*-0.0199*<br>*-0.0034*<br>*-0.0925*<br>-0.0329<br>-1<br>0.38<br>0.74<br>0.63	EQ-5D<br>SF-36<br>SF-36<br>SF-36<br>SF-36<br>EQ-5D<br>TTO<br>EQ-5D<br>EQ-5D<br>EQ-5D	Separate other-cause mortality (UK age- and sex-specific mortality rates) and deaths caused by stroke and drug toxicity.	3.5%.	McKenna 2009[28]
**Hip osteoarthritis†** (Hip replacement vs. non-operative approach, mean age 63 years)	Before surgery<br>12 months after surgery	0.805<br>0.858	15D	Quality-adjustment weights before and 12 months after surgery used for extrapolation over the remaining life expectancy to yield the QALY gain. Years of life lost were zero. We calculated total QALYs.	3%.	Rasanen 2007[29]
**Rheumatoid arthritis‡** (TNF inhibitor + methotrexate vs. methotrexate, mean age 55 years)	Conversion from HAQ score to QALYs by formula (gradual increase in HAQ to reflect disease progression)	0.862-0.327HAQ §	EQ-5D	Background mortality from standard UK life tables. To reflect increased mortality in RA patients, a relative risk of 1.33 per unit HAQ was applied.	1.5%.	Chen 2006[30]
**Acute stroke*** (Stroke unit vs. general ward, age 70 years)	Mild sequelae<br>Moderate sequelae<br>Severe sequelae	0.783<br>0.612<br>0.468	EQ-5D<br>EQ-5D<br>EQ-5D	Separate deaths from all causes (background mortality in the Norwegian general population) and deaths from CVDs. Norwegian data on incidence of cardiovascular events.	4%.	Hamidi 2010[31]

* Markov model.

† Simple patient care pathway.

‡ Discrete event simulation.

§ The quality-adjustment weight calculated from the equation gives negative values at HAQ 2.75 or higher. This results from applying linear regression to a data set of HAQ and EQ-5D scores from actual RA patients, some of whom gave EQ-5D responses which map to negative quality-adjustment weights using the standard UK tariff (P. Barton 2011, personal communication). This results in few average QALYs when the quality-adjustment weights are summed over time. Rheumatoid arthritis patients with an average age of 55 yield surprisingly few remaining QALYs (6.1), which results in few lifetime QALYs and a high proportional shortfall.

Abbreviations: AADs, antiarrhythmic drugs; AF, atrial fibrillation; BMI, body mass index; DMARD, disease-modifying antirheumatic drug; EQ-5D/HUI/SF-36/15D, generic measures of health outcome; HAQ, health assessment questionnaire used as a measure of health outcome for rheumatoid arthritis; NA, not available; NSR, normal sinus rhythm; RA, rheumatoid arthritis; RFCA, radiofrequency catheter ablation; RY, roux-en-y; SAH, subarachnoid haemorrhage; SG, standard gamble; TNF, tumour necrosis factor; TTO, time trade-off; VAS, visual analogue scale.
