# Supplementary material for: Making use of equity sensitive QALYs: a case study on identifying the worse off across diseases
Source: Cost Eff Resour Alloc. 2014 Jul 23;12:16. doi: 10.1186/1478-7547-12-16 (PMC4118218; doi:10.1186/1478-7547-12-16)
Supplement: Additional file 1 — Search strategies. [file 1478-7547-12-16-S1.pdf]

Additional file 1

Primarily, we accessed HTA project databases at the Norwegian Knowledge Centre for the Health Services and other HTA agencies in countries with a similar health system, especially the Nordic countries and the NICE/NIHR HTA programme in the UK. Examples of health technology assessment agencies:

CADTH – Canadian Agency for Drugs and Technologies in Health. www.cadth.ca

DACETHA – Danish Centre for Health Technology Assessment. www.dacehta.dk

DIMDI – German Agency for HTA at the German Institute for Medical Documentation and Information. www.dimdi.de

FinOHTA – Finnish Office for Health Technology Assessment. www.finohta.fi

NETSCC, HTA – NIHR Coordinating Centre for Health Technology Assessment. www.hta.ac.uk, www.nice.org.uk

NOKC – Norwegian Knowledge Centre for the Health Services. www.kunnskapssenteret.no

SBU – Swedish Council on Technology Assessment in Health Care. www.sbu.se

ZonMw – The Medical and Health Research Council of the Netherlands. www.zonmw.nl, www.cvz.nl

Source: www.inahta.org

Literature search carried out in March/April and November/December 2011 for qualified articles in selected international databases. In the course of the search for relevant studies, the criteria in box 1 were identified as necessary and sufficient to estimate the lifetime QALYs as described in the article.

Box 1:

Checklist: inclusion criteria for studies on condition-intervention pairs

- Clinical effectiveness is reported in terms of mean incremental Quality-Adjusted Life Years ($QALY_{gain}$).
- Expected QALYs with standard care ($QALE_{std}$) are reported, or available data on quality-adjustment weights and life expectancy with the condition are reported so that QALYs can be estimated.
- Lifetime horizon is used.
- Average age of the patients is reported.
- Undiscounted data is reported or available upon request.
- Standard care is used as the comparator.
- There are comparable levels of background mortality and general standard of health care.

The search strategy does not comply with current standards of a complete systematic literature review; we are aware that relevant studies might have been ignored. The quality of the studies was not assessed.

Listed in order of priority, i.e., we did not proceed to the next step if literature appropriate for inclusion in our analysis was identified:

1) The Cochrane Library (CDSR, DARE, HTA and NHSEED)
2) CRD
3) Pubmed, Embase
4) ISI Web of Science
5) Google Scholar.

Search terms used for conditions and interventions:

Condition	Intervention
Morbid obesity, obesity	Bariatric surgery, gastric bypass
Hip osteoarthritis	Hip replacement, hip arthroplasty
Atrial fibrillation	Catheter ablation, ablation
Deafness	Cochlear implantation
Stroke	Stroke unit, stroke management
Rheumatoid arthritis	TNF inhibitor
Depression	Therapy
Opioid dependence	Maintenance therapy
Multiple sclerosis	Treatment
Attention deficit hyperactivity disorder(ADHD)	Methylphenidate
Cerebral aneurysm	Coiling

We did not identify eligible studies reporting expected QALYs in a lifetime horizon for depression, opioid dependence, multiple sclerosis, and ADHD. These cases were exluded from the analysis.

We identified one non-economic evaluation that estimated quality-adjusted life expectancy (QALE) and loss of QALE for one of the selected diseases (Lee HY, Hwang JS, Jeng JS, Wang JD. Quality-adjusted life expectancy (QALE) and loss of QALE for patients with ischemic stroke and intracerebral hemorrhage: a 13-year follow-up. *Stroke* 2010;41(4):739-44).

Search strategy:

Cochrane Library (CDSR, DARE, HTA, NHSEED):

Search field: search all text.
1 quality-adjusted life years
2 qaly
3 #1 or #2
4 Intervention
5 #3 AND #4
6 Condition
7 #3 AND #6
8 #5 AND #6

CRD:
Search field: any field
1 Intervention
2 Condition
3 #1 and #2

Pubmed and Embase:

Search field: all fields
1 quality-adjusted life years
2 qaly
3 #1 OR #2
4 Condition
5 #3 AND #4
6 Intervention
7 #3 AND #6
8 #5 AND #7

ISI Web of Science
Search field: topic
1 quality-adjusted life years
2 qaly
3 #1 or #2
4 Intervention
5 #3 AND #4
6 Condition
7 #3 AND #6
8 #5 AND #7

Google scholar:

Condition AND qaly OR quality-adjusted life years

Intervention AND qaly OR quality-adjusted life years
